# Editorial: Combinatory Approaches of Epigenetic Regulators in T Cell-Based Immunotherapy

**DOI:** 10.3389/fgene.2022.914907

**Published:** 2022-06-07

**Authors:** Jingying Zhou

**Affiliations:** School of Biomedical Sciences, The Chinese University of Hong Kong, Hong Kong SAR, China

**Keywords:** immunotherapy, epigenetics, CD8+T cell, multi-omics analysis of cancer, biomarker for immunotherapy response

T cell-based immunotherapy, which developed based on the understanding of T-cell capacity for antigen-directed cytotoxicity, has emerged as a powerful tool in the armamentarium against cancer (Ribas and Wolchok 2018). Nevertheless, recent challenges include discovering why immunotherapy works so dramatically in some patients but not in others, and how to design a multipronged approach for a complete curative regimen in a patient-specific manner. Accumulating evidence has highlighted the importance of epigenetic mechanisms in determining T-cell fates and the corresponding features of immunotherapy-responsive T cells, while epigenetic drugs have been demonstrated to improve cancer immunotherapy efficacy in many aspects (Liu et al., 2022). In this Research Topic “*Combinatory approaches of epigenetic regulators in T cell-based immunotherapy*”, a diverse range of examples are included that cover methodological approaches of multi-omics analysis and biomarker exploration, together with clinical studies and literature reviews of research progress made in current epigenetic and immunotherapy combinatory approaches.

Chimeric antigen receptor T (CAR-T) immunotherapy, which utilized genetically modified autologous T cells, has shown promising results in treating patients with blood cancers. The paper by Zhou et al. describes a clinical trial study of anti-CD19 CAR-T cell therapy in patients with relapsed/refractory B-cell lymphoma. Besides therapeutic benefit, they demonstrated that the side effects caused by this CAR-T cell therapy can be successfully managed by administering interleukin 6 antagonist, glucocorticoids, and/or plasmapheresis, which provides an effective and safe treatment regimen for B-cell lymphoma (Zhou et al.).

Leveraging the power of technological advances in molecular biology, recent multi-omics studies, including single-cell RNA sequencing, single-cell assay for transposase-accessible chromatin (ATAC) sequencing, and single-cell chromatin immunoprecipitation (ChIP) sequencing, have provided mechanistic insights into understanding the features of immunotherapy-responsive or resistant T cells, i.e., in T-cell fate determination (Grosselin et al., 2019). Combining analysis of single-cell RNA sequencing, proteomics, and metabolomics, You et al. provided an example of identifying new pathways and molecules in limited numbers of patient-derived CD8+T cells. Compared to CD8+T cells from healthy donors, their data implied that the complex transcriptomic and metabolic changes could determine the T-cell activation status and function in patients (You et al.).

T cells undergo extensive epigenome remodeling in response to different microenvironmental cues. Epigenetic modifications *via* non-coding RNAs, DNA methylation, and histone modifications are demonstrated to have a strong effect on the phenotypic stability and anti-tumor function of CD8+T cells, leading to varied response rates and therapeutic resistance of T cell-based immunotherapy. One comprehensive review paper in this issue summarizes the current understanding of epigenetic modifications in determining CD8+T-cell fate in cancers. They describe the epigenetic enzymes and intracellular microRNAs in imprinting T-cell epigenomes that drive T-cell functional exhaustion. They also discuss the potential epigenetic interventions to rescue T-cell function for combinatory immunotherapy approaches in cancers (Wong et al.). In addition, the paper provided by Carlos-Reyes et al. focuses on non-coding RNAs, in particular circular RNAs (circRNAs), in regulating T-cell functions in response to cancers (Carlos-Reyes et al.). This knowledge collectively implies that regulating the complex epigenome of T cells may provide translational implications to advance current immunotherapy for cancer treatments.

The importance of developing novel non-invasive biomarkers in monitoring the immunotherapeutic response in patients should also be noted. The recent emergence of quantitative imaging biomarkers, like *in vivo* imaging technologies that interrogate T-cell responses, metabolic activities, and the immune microenvironment provides promising opportunities. One aspect of *in vivo* imaging utilizing novel specific positron emission tomography (PET) probes to target key molecules related to immunotherapy also offers a powerful tool to monitor patient response (Nisar et al., 2020). For example, the parameters derived from 2-Deoxy-2-(18F)-fluoro-D-glucose (18F-FDG) positron emission tomography/computed tomography (PET/CT), which takes advantage of non-invasively evaluating the glucose metabolic levels, are demonstrated to be correlated with the treatment response of immune checkpoint inhibitors in patients with non-small cell lung cancer (NSCLC) (Liao et al.).

In conclusion, the burgeoning field of cancer immunotherapy continues to grow as indications for currently approved combinatory therapies expand and the search for novel druggable targets continues. Further investigations into the molecular mechanisms of fate determinations for T cells and also other immune cells will allow us to achieve durable responses and survival benefits for the majority of patients with different cancers.
